# Label-Free Enrichment of Highly Metastatic Tumor-Initiating Cells up to a Monoclonal State

**DOI:** 10.34133/bmr.0168

**Published:** 2025-04-02

**Authors:** Larissa M. Ciaramicoli, Haw-Young Kwon, Chun Y. Im, Namhui Kim, Yoojin Oh, Young-Tae Chang, Nam-Young Kang

**Affiliations:** ^1^ Department of Chemistry, Pohang University of Science and Technology, Pohang 37673, Republic of Korea.; ^2^ SenPro Inc., Pohang University of Science and Technology, Pohang, Gyeongbuk 37673, Republic of Korea.; ^3^ New Drug Development Center, Daegu-Gyeongbuk Medical Innovation Foundation (K-MEDIhub), Daegu 41061, Republic of Korea.; ^4^ Department of Convergence I.T. Engineering, Pohang University of Science and Technology (POSTECH), Pohang 37673, Republic of Korea.

## Introduction

Cancer stem cells (CSCs) are a subpopulation of cancer cells with self-renewal capabilities, tumor initiation properties, and differentiation abilities, which drive tumor growth, metastasis, and recurrence [1,2]. Depleting CSCs in tumors has been shown to inhibit cancer metastasis, implicating their primary role in cancer resistance [3–6]. Therefore, targeting CSCs may be key to preventing tumor spread.

Most tumor cells cannot form a new tumor when transplanted into a nontumor site or another body. The ability of CSCs to generate new tumors shares some features with embryonic stem cells, but they have different appearances [7,8]. To avoid a possible misconception, the term CSC has been replaced with tumor-initiating cells (TICs).

Identifying and isolating TICs has proven challenging because they do not have a standard biomarker and mostly overlap with normal stem cell markers. Therefore, multidimensional techniques are required for their isolation: a fluorescent approach, such as immunochemistry, side population assay, or fluorescent probes, followed by 3-dimensional sphere culture [9–18].

Culturing TICs using a sphere formation assay is conducted under nonadherent and serum-free conditions [19], allowing for cell-to-cell attachment while leading differentiated tumor cells to undergo apoptosis [20]. However, the method does not guarantee the purity of the cells [21]. Depending on the application of TIC analysis in vitro, a purified population may be required. We propose a label-free method to enrich an aggressive TIC population, which shows high expression levels of TIC/metastasis markers (CD44 and CD54), and demonstrate improved tumor formation as well as metastasis in lung and thyroid cancer in xenograft models.

## Materials and Methods

### Cell culture

Non-small-cell lung cancer TS32 cells (labeled in this paper as TS32-R0) were obtained in partnership with Dr. Bing Lim, using sphere-forming CD166+ TICs. The standard media for TS32-R0 consisted of the following: Dulbecco’s modified Eagle medium (DMEM)/F12 medium (Gibco) supplemented with 2 mM l-glutamine (Gibco), 15 mM Hepes (Gibco), 1% nonessential amino acids (Gibco), 1% antibiotic–antimycotic (Gibco), 4 mg/ml of bovine serum albumin (BSA; Invitrogen), 5 ml of insulin/transferrin/selenium acid (BD Biosciences), 50 ng/ml epidermal growth factor (Invitrogen), and 20 ng/ml basic fibroblast growth factor (Invitrogen).

### Single-cell preparation

Cultured spheroids were centrifuged, and the pellet was suspended in 50 μl of StemPro Accutase Cell Dissociation Reagent (Gibco), pipetted, and incubated at room temperature (RT) for 1 min. After washing the cells with phosphate-buffered saline (PBS), the pellet was suspended in standard media and passed through a 40-μm strainer (Corning).

### Morphology-based spheroid assay

TS32-R0 single cells (1 × 10^5^) were seeded into a 10-cm petri dish and allowed to grow in spheroids for 7 d. The heterogeneity of the cell population was observed with a bright-field microscope by EVOS FL. Well-formed rounded spheres sized between 100 and 200 μm were selected with a mechanical pipette. Spheres were made into single cells, and the same amount was seeded into a 10-cm dish for the next round. For better comprehension, picked cells were labeled according to the round of selection (example: TS32-R9 refers to the ninth round of picking).

### Antibodies and reagents

The vendor from which each antibody was purchased is listed in Table S1. The unconjugated antibodies (Invitrogen) and Maxpar Ready antibodies (BioLegend, San Diego, CA) were labeled with metal tags using Maxpar X8 Polymer Kits (Fluidigm, South San Francisco, CA, USA) according to the manufacturer’s instructions.

### Mass cytometry

Samples were stained with 5 μM cisplatin (Fluidigm, South San Francisco, CA, USA) in RPMI serum-free media to distinguish live–dead cells, followed by washing with Maxpar cell staining buffer (Fluidigm, South San Francisco, CA, USA). After centrifugation, the cells were resuspended in Maxpar cell staining buffer (Fluidigm, South San Francisco, CA, USA) at a concentration of ~3 × 10^6^ cells/ml for antibody staining. After 30-min staining with an antibody cocktail (surface marker antibodies), all samples were washed with Maxpar cell staining buffer. Subsequently, they were fixed in 1.6% formaldehyde (Thermo Fisher Scientific, Rockford, IL, USA) diluted in PBS for 10 min. Right after, the cells were treated with 125 nM DNA intercalator dissolved in Maxpar Fix and Perm buffer (Fluidigm, South San Francisco, CA, USA) according to the manufacturer’s recommendations. After multiple washings with Maxpar cell staining buffer, the cells were resuspended with antibodies for intracellular marker protein. After multiple washings with Maxpar cell staining buffer, the cells were resuspended with Milli-Q water containing 0.1× EQ Element Calibration Beads (Fluidigm, South San Francisco, CA, USA). Before mass cytometry, cells were filtered through a 35-μm nylon mesh (BD Biosciences, MA, USA) and sampled at a rate of 300 to 500 cells/s using a Helios mass cytometer (Fluidigm, South San Francisco, CA, USA).

### Mass cytometry data analysis

After bead-based normalization in CyTOF software version 6.7, Flow Cytometry Software (FCS) files were uploaded to Cytobank 7.2 (Cytobank, Inc.) with Cytobank default arcsinh transformation (scale factor 5). FCS files of CyToF were preprocessed by manual cellular events gating based on DNA intercalators, ^140^Ce EQ. Bead and cisplatin data were used to remove debris, doublets, normalized beads, and dead cells. The data analysis was conducted using visualization for stochastic neighbor embedding (viSNE) with equal sampling for each comparison, a perplexity of 30, a theta value of 0.5, and 1,000 iterations. For the viSNE analysis, event sampling consisted of 11,111 cells per sample, with 5,000 iterations, resulting in a final Kullback–Leibler divergence of 3.44.

### TS32-TiY+ cell sorting

TS32-R0 was made into single cells and stained with tumor-initiating probe yellow (TiY; 100 nM) for 30 min, at RT. Cells were washed, and the pellet was resuspended in standard media before flow cytometry. Live cells gated with side scatter area vs. forward scatter area (FSC-A), refined with the single-cell gating, forward scatter height vs. FSC-A, were read by 10,000 cell events (excitation: 496 to 554 nm/emission: 576 to 586 nm); 10% of the population was sorted by the S3e cell sorter (Bio-Rad). All TS32-TiY+ samples were prepared before experiments.

### In vivo tumor formation and size evaluation

Subcutaneous injections were administered in the lower abdominal region of 6-month-old NOD scid gamma (NSG) mice. TS32-R0, TS32-TiY+, and TS32-R9 were suspended in high-glucose DMEM (Welgene, LM 001-05) as single cells. A volume of 100 μl of media containing 5 × 10^5^ cells was injected per site of injection (2 sites/mice). The experiment was made in triplicate (*n* = 3) per cell type. After 4 weeks, mice were sacrificed, and tumors were extracted and pictured for size evaluation. ImageJ was used for quantitative analysis of the average length (cm), and the volume was calculated to the approximate volume of a sphere (cm^3^).

### Metastasis model

Intravenous injections were administered to 8-week-old NSG mice. TS32-R0, TS32-TiY+, and TS32-R9 were suspended as single cells in high-glucose DMEM (Welgene, LM 001-05). A volume of 100 μl of media containing 1 × 10^5^ cells was injected into the eye of mice (1 injection/mice). The experiment was made in triplicate (*n* = 3) per cell type. The weight was monitored weekly to avoid more than 20% weight loss. After 8 weeks, mice were sacrificed, and the organs were extracted and immediately frozen for metastasis investigation.

### Tissue imaging

Tissue section images were obtained using fluorescent microscopy (Axio Observer, ZEISS, Germany). The frozen tissues were sectioned by a cryostat (Leica CM1850) with 10-μm thickness, and sections were attached to poly-l-lysine-coated slides. Organs were harvested and cryo-sectioned, followed by hematoxylin and eosin (H&E) staining. For H&E staining, the tissue was counterstained for 5 min. To turn the stain blue, slides passed through 4 changes of tap water, 5 min each. For immunohistochemistry, the tissue slides were washed with PBS to remove the optimal cutting temperature compound. Samples were stained with CD54 antibody (Invitrogen, 14-0549-82) diluted in PBS (1% BSA) for 30 min, at RT. After washing in PBS, tissue samples were incubated with goat anti-mouse immunoglobulin G (H+L) Highly Cross-Adsorbed Secondary Antibody (Invitrogen, A32723, conjugated with Alexa Fluor Plus 488) diluted in PBS (1% BSA) for 30 min, at RT. Then, the section was incubated with Hoechst for 10 min at RT.

## Results and Discussion

### The enrichment tool of TICs: Morphology-based spheroid assay

As mentioned before, besides several techniques for TIC isolation, the cell population is not homogeneous, which is detrimental to TIC-targeted therapeutic approaches. For that, we first considered sorting with TiY, the first probe to stain TIC selectively in vitro [22]. However, TiY therapeutics’ abilities gradually decrease cells’ viability, which could impair tumorspheres from growing after subsequential rounds of sorting [22,23].

In spheroid assay, controlling the shape and size is essential for cell biogenesis, survival, and expansion [24]. Tumorspheres should be solid and rounded [21,25–27]. The size should not exceed 250 μm, because big aggregates can create a cytotoxic core that impedes tumor homeostasis and creates a hypoxic environment that can lead to sphere necrosis [26,27].

**Fig. 1.**
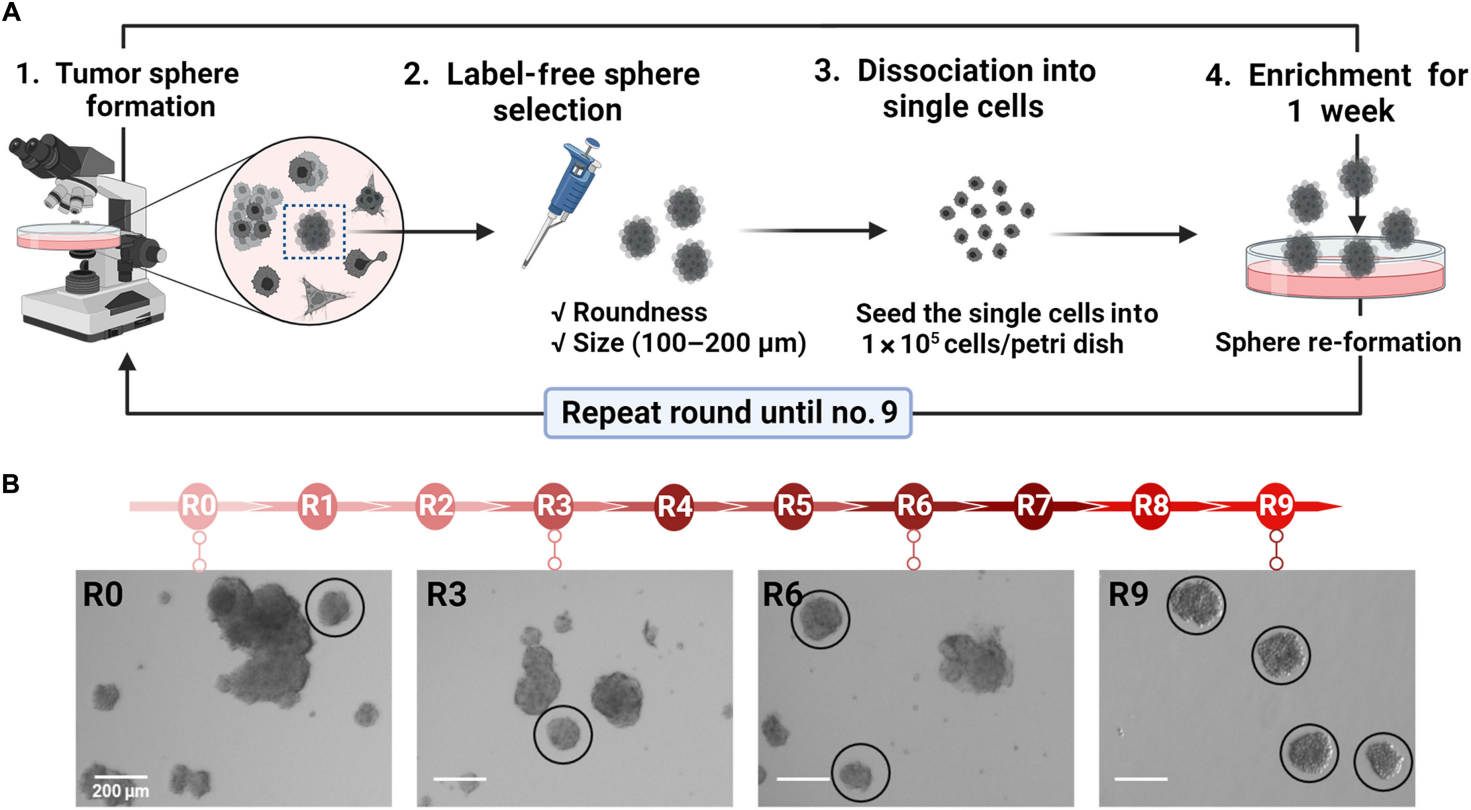
Development of the morphology-based spheroid assay (MBSA). (A) Scheme of the mechanical pipetting method for picking tumorspheres. Morphology was analyzed under the microscope by roundness and size (range from 100 to 200 μm). The best sphere forms were picked by mechanical pipetting. The spheres were dissociated into single cells to enrich the selected population and allowed to grow/expand in spheroids for 1 week. The cells were further analyzed and purified through repeated picking selection until homogeneity was achieved. (B) Tumorsphere formation through the rounds. The bright-field images show sphere morphology optimization by picking, until the establishment of homogeneous tumorspheres in round 9. The morphological confirmation of the spheroid of each round was done through the subsequent 3 experiments.

The so-called morphology-based spheroid assay (MBSA) is a laborious work but effective for controlling tumorsphere morphology, easily accessed using a bright-field microscope (Fig. 1A). The well-rounded spheres were manually selected by mechanical pipetting, within the 100- to 200-μm range of sphere size, and seeded as single cells for a week until the next round. We monitored the morphology changes through the rounds until good homogeneity was achieved with round 9 cells, TS32-R9 (Fig. 1B).

### CyTOF analysis of high-purity TIC population

**Fig. 2.**
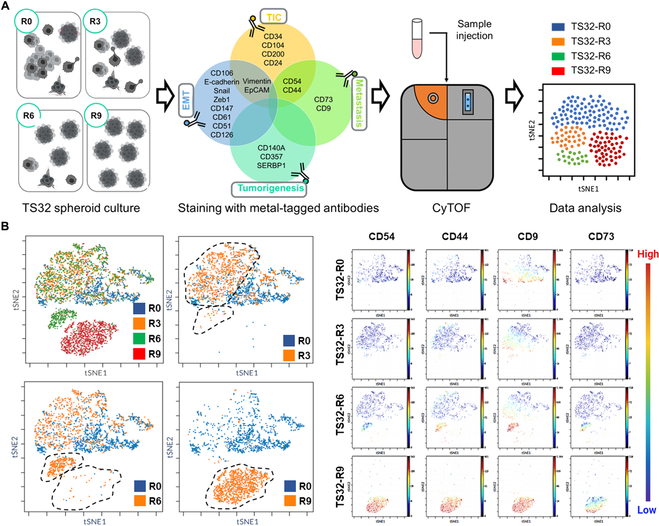
Characterization of tumor-initiating cells (TICs) with high-purity populations by cytometry by time of flight (CyTOF) analysis. (A) Schematic representation of cell preparation for CyTOF analysis. Among various rounds of cells, critical rounds such as R0, R3, R6, and R9 were characterized using CyTOF to identify TIC properties. Different rounds of cell populations were labeled with several biomarkers such as TIC, metastasis, tumorigenesis, and epithelial-to-mesenchymal transition (EMT) characteristics. Metal-tagged antibodies were used for cell staining before loading into the CyTOF machine. Subsequent data analysis enabled the identification and characterization of distinct cell clusters. (B) Left: CyTOF data analysis reveals the enrichment of different cell clusters across multiple rounds, leading to the clear distinction between TS32-R0 and TS32-R9 cell populations. Right: TS32-R9 selected cells exhibit high expression levels of TIC and metastasis-associated cell markers (CD44, CD54, CD9, and CD73) compared to the TS32-R0 cell population. EpCAM, epithelial cell adhesion molecule; SERBP1, serum response element-binding protein 1; tSNE, t-distributed stochastic neighbor embedding.

We performed cytometry by time of flight (CyTOF) analysis for phenotype characterization through the rounds, including TICs [28–31], metastasis [32–34], epithelial-to-mesenchymal transition [35–41], and tumorigenesis antibodies [42–44], while some of them intrinsically overlap [45–49] (Fig. 2A and Table S1).

The comparison shows the enrichment between rounds until the formation of a distinctive cluster. As a result, the purified TS32-R9 cell line showed a higher expression level of CD44, CD54, CD9, and CD73 than TS32-R0, which is the parental cell line. These antibodies are well-known as potential TIC and metastasis biomarkers (Fig. 2B and Fig. S1). To test TIC and metastasis abilities, we designed 2 xenograft models comparing the performance of TS32-R9, TS32-R0 (original cells), and TS32-TiY+ (−R0 cells sorted with TiY, known for TIC isolation).

### Tumor formation of TS32-R9 via a xenograft model

**Fig. 3.**
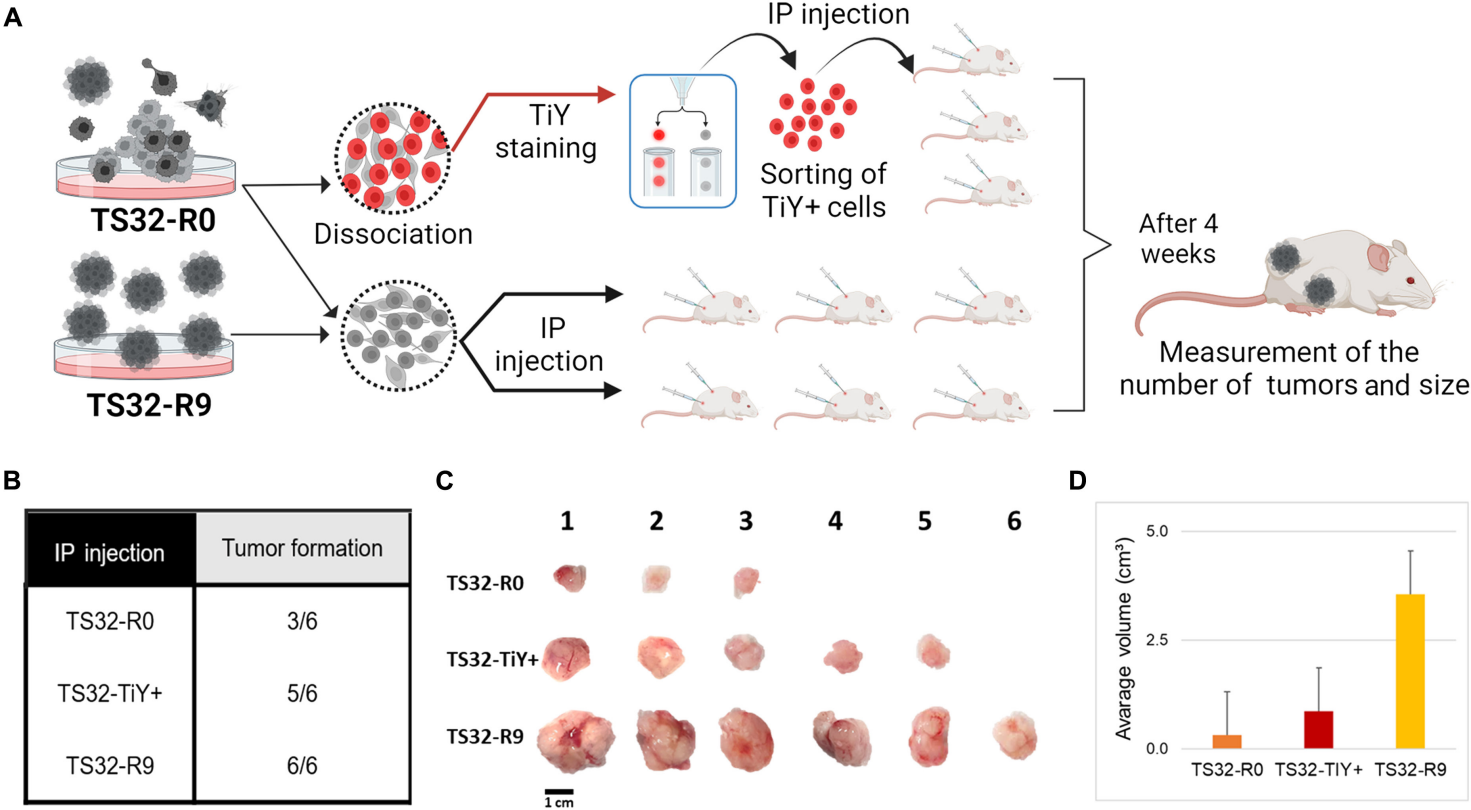
Tumor property of high-purity TIC population via a xenograft model. (A) Schematic representation illustrating the setup of a subcutaneous model to assess the tumor formation capacity of selected cells compared to TS32-R0 cells and TS32-TiY+ cells (sorted from R0). The subcutaneous model was established through subcutaneous injection at 2 distinct sites per mouse (*n* = 3), and tumor formation was visibly evident after 4 weeks. (B) Table of the number of tumors. A total of 3 mice were injected into 2 different sites, resulting in 6 injection sites. Among them, TiY-positive cells (5 tumors) and TS32-R9 (6 tumors) had better performance than R0, which formed only 3 out of 6. (C) Representative images of extracted tumors from the xenograft models allowing a size comparison between TS32-R0, TS32-TiY+ sorted cells, and TS32-R9 picked cells. (D) Quantitative analysis of the average volume of the extracted tumors (cm^3^). TiY, tumor-initiating probe yellow; IP, intraperitoneal.

A subcutaneous model was established to evaluate the ability to form tumors in vivo. Subcutaneously, single cells were injected into NSG mice, which allowed the tumor to grow for 4 weeks (Fig. 3A). Firstly, tumors were quantified by counting the number of tumors, in a total of 6 sites of injection per cell type. Only the TS32-R9 group had tumors in all sites (6/6), while the TS32-R0 group showed 3/6 tumors, and TS32-TiY+, 5/6 tumors (Fig. 3B and C). Qualitative analysis showed that TS32-R9 injected cells formed around 3-fold more giant tumors than the heterogeneous TS32-R0 and even exceeded the in vivo tumorigenic ability of TiY-sorted ones (Fig. 3D).

### Metastatic potential of high-purity TIC: TS32-R9

**Fig. 4.**
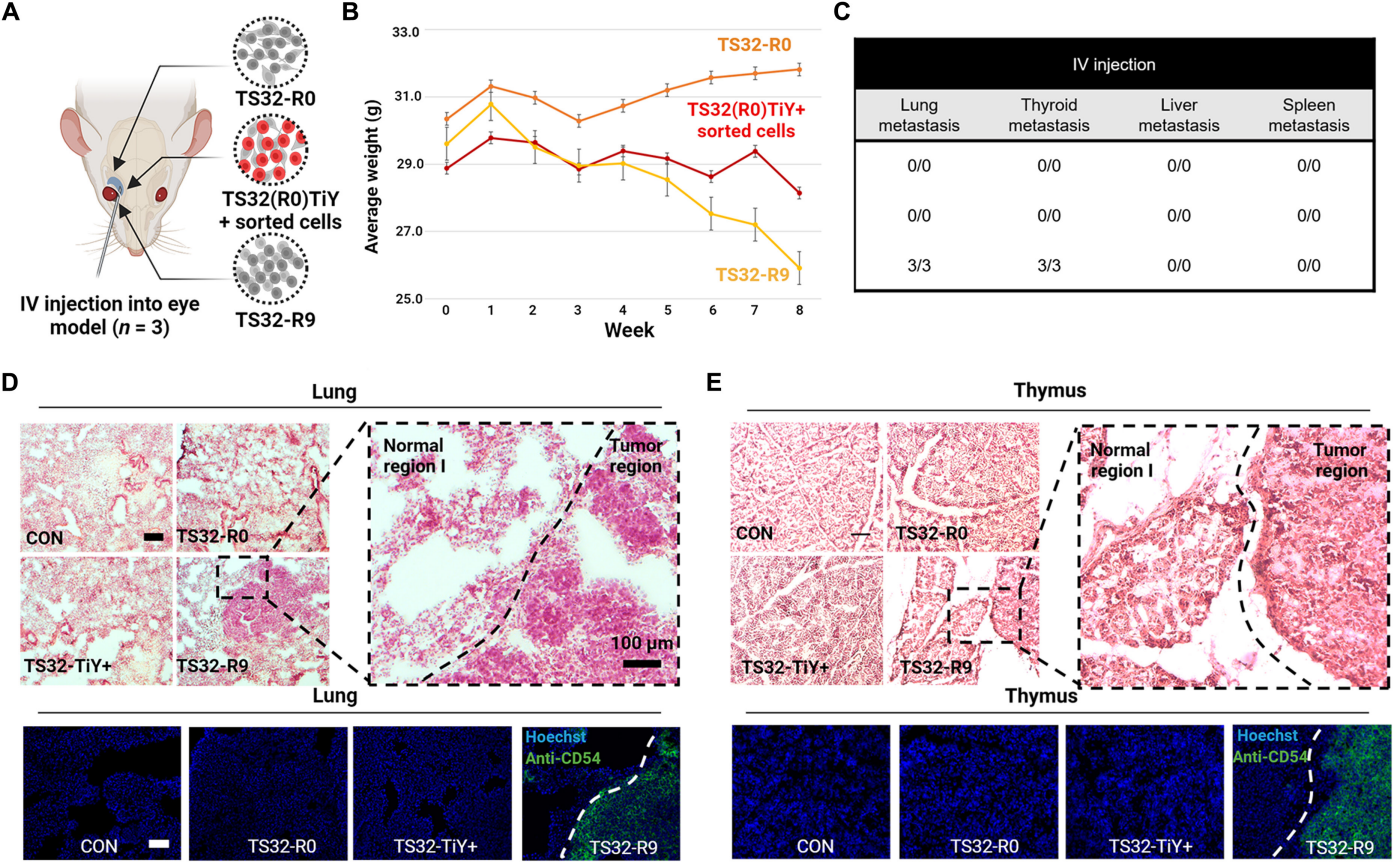
Potential of metastasis characteristics in a xenograft model with TS32-R9. (A) Schematic representation illustrating the setup of a metastasis model. Three types of cell populations were acquired through the intravenous injection of single cells into mice. (B) Evaluation of the average weight variation between all models from week 0 (before injection) and 8 weeks later (tumor extraction). (C) Quantitative data analysis. Data show the summary of the triplicate experiment metastasis check in the lung, thyroid, liver, and spleen. (D and E) Only TS32-R9 cells accrued metastasis in the lung and thyroid from all 3 mice. After 8 weeks, organs were extracted and analyzed through hematoxylin and eosin (H&E) staining. H&E staining images show the visualization of lung and thyroid tissues. Compared to a healthy control group, TS32-R0 and TS32-TiY+ (sorted from R0) cells exhibited tissue staining patterns similar to those of healthy tissues in all organs analyzed. However, TS32-R9 cells demonstrated metastasis in lung and thyroid tissues (*n* = 3). Bottom: The immunohistochemistry staining of CD54, which is a metastasis biomarker, is exclusively found in the tumor region. Data are consistently correlated with the high expression of CD54 found in the injected TS32-R9 cell line. IV, intravenous; CON, control.

Metastatic prognosis was done with an intravenous model (Fig. 4A). Mice were injected with different groups of cells, 1 site of injection/mice. While the weight was monitored weekly to avoid surpassing 20% of weight loss [50], TS32-R9 injected mice had the highest average loss (12.61%), in 8 weeks (Fig. 4B). After being sacrificed, the organs were analyzed by H&E staining. Only the TS32-R9 batch presented tumor metastasis on the lungs and thyroid, among abdomen-representative organs such as the liver and spleen. No tumor was found in the organs of TS32-R0 and TS32-TiY+ models, where the tissue disposition was like that of a healthy individual (Fig. 4C to E and Fig. S2).

We performed an immunohistochemistry experiment to determine if the tumor directly originated from TS32-R9 injected cells. The staining of the CD54 metastasis biomarker, found in TS32-R9, overlapped with the tumor found in both lung and thyroid tissues (Fig. 4D and E, bottom). These data show that TS32-R9 has a metastatic behavior, generating new tumors in lungs and thyroids compared to the TS-R0 parental cell line. The rounding culture of TICs probably leads to the enhancement of the metastatic and aggressive properties of TICs by maintaining proper size and shape. From the standpoint of molecular mechanism, TS32-R9 supports the TIC properties of aggressive tumor progression by promoting cell-to-cell interaction with high purity. This means that the parental cell line, TS32-R0, gradually loses TIC tumorigenesis characteristics such as aggressive tendency, metastasis, and invasiveness by changing into an irregularly differentiated cell morphology. Thus, the MBSA was shown as a metastatic-TIC potential method by enriching high-purity TIC without chemical aid.

## Conclusion

TIC has a vital role in cancer relapses; therefore, it is crucial to have a representative cell population for investigating chemoresistance. Purified TIC populations are difficult to isolate using immunochemistry and immunofluorescence. Here, we demonstrated an MBSA that involves morphological and physical selection of spheres based on size and shape. The results of consecutive selection in a homogeneous population indicate a more aggressive phenotype than the parental cell line.

The MBSA technique is a more manual tool based on the operator’s observation. It allows for biased selection from time to time without a physical guide, such as suitable shape and size. It is also critical to enhance TIC properties by carefully maintaining them through subculturing. Despite limitations of MBSA such as being time-consuming and labor-intensive, the enriched population of TS32-R9 retained metastatic and aggressive characteristics by generating big tumors in the lungs and thyroid in TIC with high purity. There is an issue with utilizing TICs as a cell line derived from patient tissue due to the loss of TIC purity over time. From the clinical point of view to cure cancer, there is no stable patient-derived TIC cell line capable of culturing TIC with high purity and metastatic and aggressive properties. Therefore, we suggest that the established MBSA can be utilized as a new TIC culturing method by transforming the automated cell culture system to enhance its value as an anticancer drug efficacy cell platform. This powerful method could play a potential role in organizing patient-derived tumors as a TIC cell line banking system in clinical research.

So far, several techniques have been demonstrated to isolate TICs based on cell sorting using CD makers or side populations. There is, however, no specific CD marker commercially available for detecting TICs derived from lung tumors. For example, although CD166 is known to be a CD marker for colon, lung, and prostate cancers, it is not specific to lung tumor TICs. When using Hoechst dye for side population analysis or developed TIC detection probes, there is potential chemical toxicity that can occur in isolated TICs if they are cultured for a long time to maintain TIC properties. Eventually, the simple method has the great advantage of being implemented for culturing highly purified tumorspheres with no toxicity.

Taking together, here, we have established a new label-free method called MBSA that can enrich a highly aggressive TIC population with a high expression of CD44 and CD54 metastatic makers. The purified TIC population also showed potential tumorigenesis after metastasis in the lungs and thyroid in a xenograft mouse model. To our knowledge, this finding is the first demonstration of a TIC enrichment tool based on physical properties without chemical aid, showing the high purity of TICs, including aggressive tendency, metastasis, and invasiveness.

## Ethical Approval

This research complied with all relevant ethical regulations. For this paper, NSG mice were provided by the Pohang University of Science and Technology (POSTECH) Institutional Animal Care and Use Committee (IACUC) (Approval No. 2019-0088). All mice were maintained in the animal facility of the POSTECH Biotech Center by the IACUC of POSTECH. The animal experiments were performed according to the recommended guidelines.

## Data Availability

All data generated or analyzed during this study are available from corresponding author Nam-Young Kang (knysg@postech.ac.kr) upon reasonable request.
