# Supplementary material for: Label-Free Enrichment of Highly Metastatic Tumor-Initiating Cells up to a Monoclonal State
Source: Biomater Res. 2025 Apr 2;29:0168. doi: 10.34133/bmr.0168 (PMC11964298; doi:10.34133/bmr.0168)
Supplement: Supplementary 1 — Figs. S1 and S2 Table S1 [file bmr.0168.f1.docx]

**SUPPORTING INFORMATION**

Titles

Label-free enrichment of highly metastatic tumor-initiating cells (TICs) up to a monoclonal state Label-free enrichment of metastatic TICs

**Authors**

Larissa M. Ciaramicoli,^1, †^ Haw-Young Kwon,^1,2, †^ Chun Y. Im,^3^ Namhui Kim,^3^ Yoojin Oh,^3^ Young-Tae Chang^1,2*^ and Nam-Young Kang^2,4**^

**Affiliations**

^1^ Department of Chemistry, Pohang University of Science and Technology, Pohang 37673, Republic of Korea

^2^ SenPro Inc., C5 building, Pohang University of Science and Technology, Pohang, Gyeongbuk 37673, Korea

^3^ New Drug Development Center, Daegu-Gyeongbuk Medical Innovation Foundation (K-MEDIhub), Daegu 41061, Korea

^4^ Department of Convergence I.T. Engineering, Pohang University of Science and Technology (POSTECH), Pohang 37673, Republic of Korea

^†^ These authors contributed equally to this work

^*^Correspondent author: ytchang@postech.ac.kr

^**^ Correspondent author: knysg@postech.ac.kr

Supplementary Materials

Figure S1. Completed CyTOF data analysis

Figure S2. Verification of liver and spleen tissue metastasis property – clearance of other tissues

Table S1. Antibodies utilized for CyTOF experiment


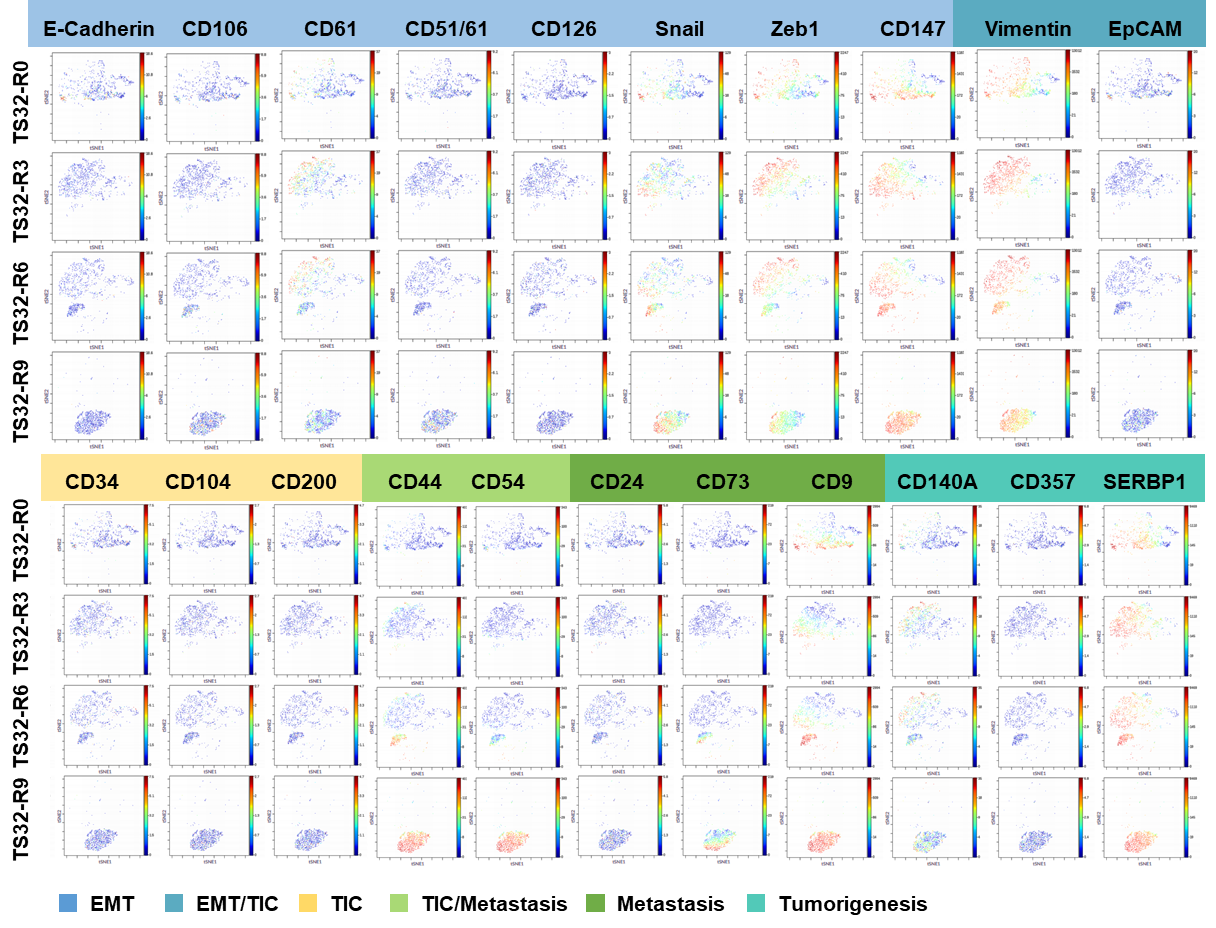


**Figure S1.** Completed CyTOF data analysis. The data shows the expression level of all the tested biomarkers comparing TIC, EMT, metastasis, and tumorigenesis-related markers in TS32-R3, -R6, and -R9 cells to those in TS32-R0 cells.


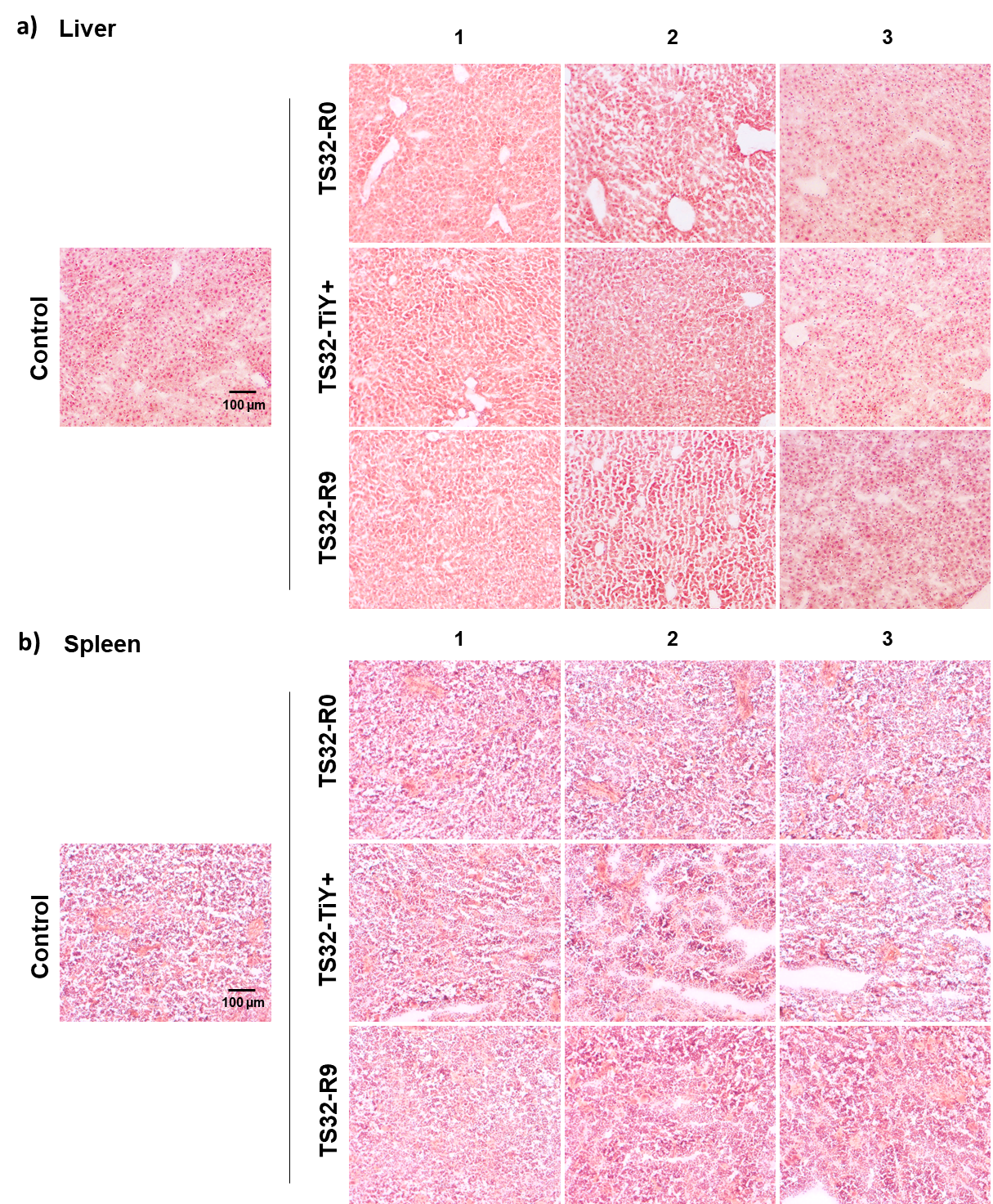


**Figure S2.** Verification of tissue metastasis properties of liver and spleen involved the clearance of other tissues. The data shows that the injection of TS32-R0, -TiY+ (sorted from R0), or -R9 sorted cells did not affect other abdominal organs, such as a) Liver and b) Spleen.


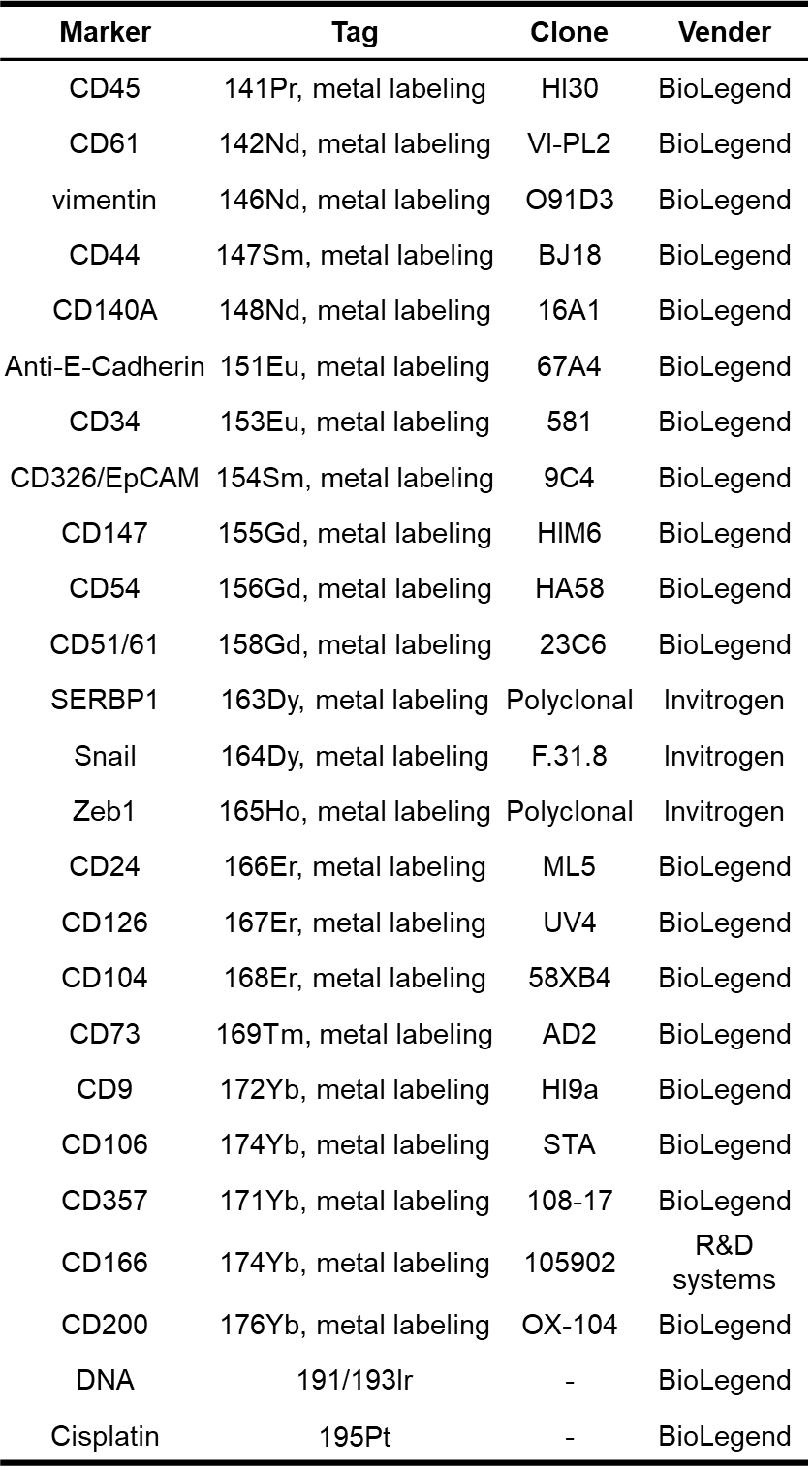


**Table S1.** Antibodies untility for CyTOF experiment.
